# Gambling Behavior and Risk Factors in Preadolescent Students: A Cross Sectional Study

**DOI:** 10.3389/fpsyg.2019.01287

**Published:** 2019-06-12

**Authors:** Nicoletta Vegni, Francesco Maria Melchiori, Caterina D’Ardia, Claudia Prestano, Massimo Canu, Giulia Piergiovanni, Gloria Di Filippo

**Affiliations:** Department of Psychology, Niccolò Cusano University, Rome, Italy

**Keywords:** gambling, risk factors, preadolescence, addiction, prevention

## Abstract

Although gambling was initially characterized as a specific phenomenon of adulthood, the progressive lowering of the age of onset, combined with earlier and increased access to the game, led researchers to study the younger population as well. According to the literature, those who develop a gambling addiction in adulthood begin to play significantly before than those who play without developing a real disorder. In this perspective, the main hypothesis of the study was that the phenomenon of gambling behavior in this younger population is already associated with specific characteristics that could lead to identify risk factors. In this paper, are reported the results of an exploratory survey on an Italian sample of 2,734 preadolescents, aged between 11 and 14 years, who replied to a self-report structured questionnaire developed *ad hoc*. Firstly, data analysis highlighted an association between the gambling behavior and individual or ecological factors, as well as a statistically significant difference in the perception of gambling between preadolescent, who play games of chance, and the others. Similarly, the binomial logistic regression performed to ascertain the effects of seven key variables on the likelihood that participants gambled with money showed a statistically significant effect for six of them. The relevant findings of this first study address a literature gap and suggest the need to investigate the preadolescent as a cohort in which it identifies predictive factors of gambling behavior in order to design effective and structured preventive interventions.

## Introduction

In recent years, addiction has undergone changes both in terms of choice of the so-called substance and for the age groups involved (Echeburúa and de Corral Gargallo, 1999; Griffiths, 2000). Although addiction is a condition associated to substance abuse disorder, it also determines other conducts that can significantly affect the lifestyle of subjects (Schulte and Hser, 2013).

In the last edition of the Diagnostic and Statistical Manual of Mental Disorders (DSM-5) (American Psychiatric Association, 2013), the pathological gambling behavior has been conceptualized differently than in previous editions, as a result of a series of empirical evidence indicating the commonality of some clinical and neurobiological correlates between pathological gambling and substance use disorders (Rash et al., 2016). The new classification into the “*Substance-Related and Addictive Disorders*” category supports the model of behavioral addictions in which people may be compulsively and dysfunctionally engaged in behaviors that do not involve exogenous drug administration, and these conducts can be conceptualized within an addiction framework as different expressions of the same underlying syndrome (Shaffer et al., 2004).

Despite the fact that in many countries gambling is forbidden to minors, in recent years, there has been a marked increase in this behavior among younger people so that from surveys conducted in different cultural contexts it emerges that a percentage between 60 and 99% of boys and between 12 and 20 years have gambled at least once (Splevins et al., 2010). The increasing number of children and underaged youth participating in games of chance for recreation and entertainment is attributable to the legalization, normalization, and proliferation of gambling opportunities/activities (Hurt et al., 2008).

Several studies have shown that the percentage of young people who gamble in a pathological way is significant and even greater than the percentage of adult pathological gamblers (Blinn-Pike et al., 2010). Using the definitions of at-risk and problem gambler that directly refer to the diagnostic criteria for pathological gambling, the review of Splevins et al. (2010) showed that a percentage of adolescents between 2 and 9% can be classified within the category of problem gamblers, while between 10 and 18% are adolescents who can be considered at-risk gamblers.

The first comprehensive review on problematic gambling in Italy noted a lack of large-scale epidemiological studies and of a national observatory regarding this issue (Croce et al., 2009). More recent studies regarding the Italian national context are now available. A survey carried out with 2,853 students aged between 13 and 20 years showed that 7% of adolescents interviewed were classified as pathological gamblers (Villella et al., 2011), while the study conducted by Donati et al. (2013) indicated that 17% of adolescents showed problematic gambling behaviors.

As far as ecological factors are concerned, the crucial role of family and play behavior of friends has been widely documented. In particular, a strong association between parents’ and children’s gambling behavior has emerged (Hardoon et al., 2004), and it has been highlighted that the spread of gambling in the group of friends influences the practice of gambling among adolescents (Gupta and Derevensky, 1998).

Traditionally, gambling in youth was considered as related to poor academic achievement, truancy, criminal involvement, and delinquency. More recently, investigators have examined the relationship between gambling and delinquent behaviors among adolescents in a systematic way, shifting the understanding beyond the explanation that delinquency associated with problem gambling is merely financially motivated by gambling losses (Kryszajtys et al., 2018). This suggests that young players may have more general problems of conduct than specific criminal behavior.

Conversely, in relation to poor academic achievement, it has been highlighted that problem gambling in adolescence affect students’ performance mainly by reducing the time spent in studying (Allami et al., 2018).

Although the phenomenon of gambling has been widely analyzed in the adult population and there are numerous studies on the adolescent population, the data in the literature suggest that gambling may be a phenomenon already present in preadolescence and needs to be analyzed. In fact, the lowering of the age of onset of problematic behaviors related to pathological gambling raises a question about the presence of gambling in preadolescents, as more exposed to the use of the Internet, smartphones, and tablets as tools that could encourage this type of conduct. A series of studies (Shaffer and Hall, 2001; Vitaro et al., 2004; Winters et al., 2005; Kessler et al., 2008) have highlighted how adult pathological players started playing significantly earlier from a non-pathological player’s chronological point of view.

Nevertheless, it has been seen in the literature as, within the population of those who start playing before the age of 15, only 25% maintain the same frequency of play even in adulthood (Vitaro et al., 2004; Delfabbro et al., 2009, 2014).

In the review by Volberg and colleagues, it was shown how teenagers tend to prefer social and intimate games, such as card games and sports betting, while only a small percentage of teenagers are involved in illegal age gambling activities (Volberg et al., 2010).

Pathological and problem players seem to be more involved in machine gambling (such as slot machines and poker machines), non-strategy games (such as bingo and lottery or super jackpot), and online games; they play in different contexts such as the Internet, school, and dedicated rooms (Rahman et al., 2012; Yip et al., 2015).

It has been seen that online gambling is particularly attractive for young people due to its extreme accessibility, the large number of events dedicated to gambling, accessibility from the point of view of the economic share invested, and the multisensory experience and high level of involvement reported by young people (Brezing et al., 2010; King et al., 2010).

Considering what is present in the literature, it is evident that the phenomenon of pathological gambling in adulthood is linked to a series of risk factors already present in adolescence. At the same time, the progressive lowering of the age at the beginning, which has been seen to be one of the main risk factors, makes it necessary to analyze the presence of the phenomenon of gambling in preadolescents, an analysis that at this time cannot count on the support of validated tools and questionnaires.

Considering that young people spend part of their time playing, it is necessary to distinguish between what is considered a game and what is considered gambling, even if not in a pathological way.

According to King et al., “gaming is principally defined by its interactivity, skill-based play, and contextual indicators of progression and success. In contrast, gambling is defined by betting and wagering mechanics, predominantly chance-determined outcomes, and monetization features that involve risk and payout to the player” (King et al., 2015).

Primarily, the objective of this study is to verify the presence, the possible extent, and the characteristics of the phenomenon of gambling as defined before in a population of preadolescents (percentage, distribution by gender) to see if the population of preadolescent players shows the same characteristics as those found in larger populations at the age level (adolescents and adults). Secondly, the study aims to verify any differences in the perception of the game between those who play and those who do not, in order to identify additional specific characteristics.

In addition, on the basis of what is highlighted in the literature with respect to the risk factors detected in adults and adolescents, the study aims to assess whether and which of these factors can be predictive of the phenomenon of preadolescent gambling.

Finally, always in line with the identification of possible prodromal factors of gambling, the study wants to analyze the differences with respect to the types of games preferred by preadolescent players to assess any similarity with what emerged in the adolescent population.

In addition, the study aims to verify whether preadolescent players show the same game-level preferences highlighted in the literature as risk factors for the development of a real game disorder (Rahman et al., 2012; Yip et al., 2015).

## Materials and Methods

The investigation followed the Ethical Standards of the 1994 Declaration of Helsinki, and the study was approved by the Departmental Research Authorization Committee of Niccolò Cusano University and the Italian Ministry of Labour and Social Policy. In a prospective study of gambling perception, behavior, and risk factors, youth aged 11 to 14 years were recruited from 47 schools situated in 18 regions of Italy. The respondents’ survey was composed by 2,734 preadolescents (1,256 female and 1,452 male), enrolled in the 6, 7, and 8 grades across all national areas (18 provinces out of 20 Italian regions).

The administration of the survey was approved by the school boards of all the institutes involved, and all parents signed the informed consent and authorization to process personal data of their children. The self-report questionnaire was proposed and filled out in the classroom during school time.

The complete questionnaire developed ad hoc by the authors for the survey is composed of 19 items, 6 related to demographic characteristics of the sample and the remaining tighter focused on gambling behaviors and information related to the context of the subject. An excerpt of all the analyzed questionnaire items is provided in the appendix to facilitate the understanding of the Likert scale administered (see Supplementary Data Sheet 4).

After data screening, which excluded incomplete/invalid questionnaires, the sample presented the following characteristics: gender, 1,312 male (53%) and 1,163 female (47%); nationality, 93% Italian and 7% others; age: *M* = 12.36, SD = 0.95, distributed in 11 years old *n* = 541 (21.9%), 12 years old *n* = 803 (32.4%), 13 years old *n* = 841 (34.0%), and 14 years old *n* = 290 (11.7%).

Gamblers were defined as individuals who showed gambling behaviors in the previous year, classified as the ones who answered “yes” to the question “In the last twelve months did you game and gamble money playing any game?”

In the first sets of analysis, data were examined to determine whether there was an association between the gambling behavior and individual or ecological factors measured on nominal, continuous, or ordinal scales. Variable dependence was assessed as appropriate using chi-square for nominal variables, *t*-test for comparing groups on two continuous variables (e.g., age), or the sound nonparametric Mann-Whitney *U* test to confront two ordinal variables (e.g., Likert 5/4-point scale from fully agree to fully disagree). The decision to apply nonparametric tests was made considering the correlational research design of the survey and the non-previously validated questionnaire as the tool for collecting data. Moreover, the utilization of nonparametric analysis gives the most accurate estimates of significance in case of non-normal data distributions and variables of intrinsic ordinal nature as the ones obtained from Likert items in the questionnaire (Laake et al., 2015).

For the same reason, a Friedman test was run to determine if there were differences in the playing rates of gamers concerning different games of chance, because this nonparametric test determines if there are differences between more than two variables measured on ordinal scales, e.g., when the answers to the questionnaire items are a rank (Conover, 1999). The different categories of game taken into account were “videopoker, slot machine e video slot,” “lotto, lottery and superjackpot,” “Scratch card,” “Sport bets,” and “Daily fantasy sports.”

The second set of analyses examined the probability of being in the category “gamblers” of the dependent variable given the set of relevant independent variables already identified in base of preliminary analysis results and substantive literature support. More specifically, the following variables measured by the questionnaire were analyzed: gender, inappropriate school behavior, parent with gambling behavior, and troubles with parent – videogame-related and gambling-related. In this perspective, model selection in the multivariate logistic regression is aimed to the understanding of possible causes, knowing that certain variables did not explain much of the variation in gambling could suggest that they are probably not important causes of the variation in predicted variable. Moreover, introduction of too many variables could not only violate the parsimony principle but also produce numerically unstable estimates due to overfitting (Rothman et al., 2008).

## Results

Individual characteristics of participants who gambled (gamblers) versus participants who did not gamble (nongamblers) are shown in Supplementary Table S1.

Gamblers were more likely males, older, and showed a higher record of inappropriate behavior at school in the past. Moreover, the parents of these students presented a higher proportion of gambling behavior and family conflicts related to playing videogames or gambling. As shown in Supplementary Table S2, the two groups also differed significantly on the variable “online gambling without money.”

Subsequently, several Mann-Whitney *U* tests were run to determine if there were differences in the perception of many gambling’s facets (measured through self-report scores) between gamblers and nongamblers. To analyze the perception of the game and any differences between players and nonplayers have been isolated four variables measured through the following items: “loosing money because of gambling,” “becoming rich through gambling,” “gambling is funny,” “gambling is an exciting activity.” The distributions of the perception scores for gamers and not gamers on these four items were similar, as assessed by visual inspection. Median perception of gambling as a risk was statistically significantly lower in gamblers (3) than in nongamblers (4), *U* = 344, *z* = −4.59, *p* < 0.001, as well as the difference between median perception scores of gambling as an habit was statistically significantly lower in gamblers (3) than in nongamblers (4); *U* = 357, *z* = −3.48, *p* < 0.001. Statistically significant differences were also found between the median perception scores of gamblers and nongamblers on the variable “*losing money because of gambling*” [lower in gamblers (3) than in nongamblers (4); *U* = 327, *z* = −6.27, *p* < 0.001] and “*becoming rich through gambling*” [higher in gamblers (2) than in nongamblers (1); *U* = 519, *z* = 9.879, *p* < 0.001].

Differently, on two similar items regarding the perception of gambling as an entertaining activity and as an exciting activity, the distributions for gamblers and nongamblers were not similar, as assessed by visual inspection. One of the two items concerned the perception of gambling as an entertaining activity; the Mann-Whitney *U* test revealed that scores for gamblers (mean rank = 1.8) were significantly higher than for nongamblers (mean rank = 1.14; *U* = 608, *z* = 17.52, *p* < 0.001). The last item concerned the perception of gambling as an exciting activity; the Mann-Whitney *U* test revealed that scores for gamblers (mean rank = 1.7) were significantly higher than for nongamblers (mean rank = 1.16; *U* = 569, *z* = 14.23, *p* < 0.001).

For this reason, a Friedman test was run to determine if there were differences in the playing rates of gamers concerning different games of chance, because this nonparametric test determine if there are differences between more than two variables measured on ordinal scale, i.e., when the answers to the questionnaire items are a rank (Conover, 1999). The students who stated to have gambled money in the previous 12 months were asked in the following question about the frequency they played different group of games.

Pairwise comparisons were performed (IBM Corporation Released, 2017) with a Bonferroni correction for multiple comparisons. Gambling/playing rate was statistically significantly different in the five groups of games, *χ*^2^ (4) = 226.693, *p* < 0.0005. The values of post hoc analysis are presented in Supplementary Table S2, and the Pairwise Friedman’s comparisons revealed relevant statistically significant differences in playing rates of gamers. In fact, the category of game of chance constituted by “videopoker, slot machine e video slot” (mean rank = 2.46) is preferred to all other kinds of game of chance, except “lotto, lottery and superjackpot” (mean rank = 2.50). In the case of “Lotto, lottery, SuperJackpot,” this category of game of chance is preferred to “Scratch card” (mean rank = 3.30) in a statistically significant way, but it is also statistically less played in comparison to “Sport bets” (mean rank = 3.35) and “Daily fantasy sports” (mean rank = 3.40). None of the remaining differences were statistically significant.

Regarding the second set of analyses, Supplementary Table S3 provides the model used in the binomial logistic regression performed to ascertain the effects of key variables on the likelihood that participants played game of chance with money. The logistic regression model was statistically significant, *χ*^2^ (7) = 326, *p* < 0.001. The model explained 23.0% (Nagelkerke *R*^2^) of the variance in the predicted variable (gambling behavior) and demonstrated a percentage accuracy in classification (PAC) equal to 86.6%. Sensitivity was 22.5%, specificity was 97.6%, positive predictive value was 62.2%, and negative predictive value was 87.9%. Of the seven predictor variables only six were statistically significant: gender, inappropriate school behavior, parents with gambling behavior, troubles with parents – videogames related, online gambling without money, and age (as shown in Supplementary Table S3). Analysis showed that male had 2.96 times higher odds to be gamers than females (OR = 0.337; 95% CI 0.248–0.458), and increasing age was associated with an increased likelihood of gambling behavior. Also, inappropriate school behavior (OR = 1.859; 95% CI 1.395–2.477), parents with gambling behavior (OR = 3.836; 95% CI 2.871–5.125), troubles with parents – videogames related (OR = 1.285; 95% CI .510–3.236), and online gambling without money (OR = 2.297; 95% CI 1.681–3.139) increased the likelihood of gambling. By contrast, the “Troubles with parents – gambling related” variable was not statistically significant, probably because of the extremely unbalanced case ratio between the two modalities.

## Discussion

The first objective of this study was to evaluate the presence or absence and the consequent extent of the phenomenon of gambling in a population of preadolescents and to understand which factors are associated to the progressive lowering of the age of onset.

Consistently with the literature on the adult and adolescent population, the evidence presented thus far supports the idea that even in the preadolescent population players tend to be predominantly males (Hurt et al., 2008; Splevins et al., 2010; Villella et al., 2011; Dowling et al., 2017).

One of the more significant findings to emerge from this study is that players of game of chance have a significantly different perception of the game than nonplayers, i.e., they see the game as “less risky” and perceive less risk of losing money through the game. In addition, confirming this “altered” perception, they show higher values than nonplayers in the perception of being able to become rich through the game (Hurt et al., 2008; Dowling et al., 2017). Gamblers have a perception of the game as exciting and fun, a tendency which increases with age. This pattern seems to confirm what is expressed in the literature regarding the theme of sensation seeking and its connection with the development of gambling disease (Dickson et al., 2002, 2008; Hardoon and Derevensky, 2002; Messerlian et al., 2007; Blinn-Pike et al., 2010; Shead et al., 2010; Ariyabuddhiphongs, 2011; Lussier et al., 2014).

Even more importantly, some possible predictive factors of gambling emerged among the variables analyzed: thus, the phenomenon of gambling was associated with problems of school conduct, problems with parents related to the use of video games and, interestingly, also to the presence of parents who are gamers.

Since there are no validated tools in the literature for the diagnosis of preadolescent gambling, the analyses were conducted on those who were “gamblers” according to what was previously stated. It is therefore of particular relevance that the sample of preadolescent gamblers shows descriptive characteristics and predictive factors similar to those highlighted by the literature on adolescent gamblers with a diagnosis of gambling.

In this sense, the analysis of the most frequently used game types is particularly important.

With respect to the game categories analyzed, with the exception of “Lotto, lottery, SuperJackpot,” the category that is most frequently chosen by the sample of gamblers is that of “videopoker, slot machine e video slot.”

These data are of particular relevance considering that some studies in the literature have shown that adult pathological players have shown in previous ages a strong preference for these types of games. Although it is necessary to investigate with further studies the reasons underlying the choice of this type of game by preadolescents, this fact suggests that the phenomenon of preadolescent gambling has a number of aspects and characteristics common to those identified by the literature in the analysis of the precursors of pathological gambling.

## Conclusion

There are some issues to take under consideration in framing the present results. Regarding the sample, although the numerous participants and the geographical representativeness of the population, the sample was not randomly selected. Therefore, we cannot exclude that subjects were unbalanced on unobserved, causally relevant concomitants. Although the methodology allows prediction, it should be noted that causality cannot be established from this survey, because the research design does not properly establish temporal sequence. In addition, only self-report measures and not thoroughly validated scales were used, as the objective of this study was to conduct an exploratory survey on the characteristics of the phenomenon, and there were some dichotomous variable with uneven case ratios. Furthermore, some constructs related to gambling behavior (e.g., impulsivity) and neurocognitive functioning were not analyzed in designing this first study; although in the wider research program, it is intended to explore also these factors.

Notwithstanding these limitations, the present study makes some noteworthy contributions to the understanding of the phenomenon of gambling and its characteristics in a population (preadolescents) which is still not very explored in the literature.

In particular, one significant finding is that the lowering of the age has not substantially changed what has been established in the literature with respect to the phenomenon in adolescents: the characteristics of players in terms of gender are substantially unchanged in the comparison between adolescents and preadolescents.

Moreover, from the analyses carried out, it appears that those that the literature has highlighted as risk factors of gambling in adolescence and adulthood are already present in younger players and may be predictive factors of gambling conduct already in preadolescence.

The data show, moreover, that the perception of gambling for those who play is significantly different from those who do not play, and specifically on aspects related to attractiveness, the low perception of risk and the possibility of getting rich easily. Finally, even with respect to an analysis carried out on different types of games, what emerged from the literature as additional risk factors for adolescents and adults is already present in preadolescence.

The findings of this study focus on the need to investigate the preadolescent age group in order to identify specific predictive factors of gambling in order to structure effective and structured preventive interventions and the parallel need to structure a standardized tool for the diagnosis of gambling in this specific population.

## Data Availability

The datasets generated for this study are available on request to the corresponding author.

## Ethics Statement

The study was carried out according to the principles of the 2012–2013 Helsinki Declaration. Written informed consent to participate in the study was obtained from the parents of all children. The study was approved by the IRB of the Department of Psychology of Niccolò Cusano University of Rome.

## Author Contributions

NV and GF designed and performed the design of the study and conducted the literature searches. CD, MC, and GP provided the acquisition of the data, while FM undertook the statistical analyses. NV, CP, and FM wrote the first draft of the manuscript. All authors significantly participated in interpreting the results, revising the manuscript, and approved its final version.

### Conflict of Interest Statement

The authors declare that the research was conducted in the absence of any commercial or financial relationships that could be construed as a potential conflict of interest.

## Supplementary Material

The Supplementary Material for this article can be found online at: https://www.frontiersin.org/article/10.3389/fpsyg.2019.01287/full#supplementary-material
