# Supplementary material for: Gambling Behavior and Risk Factors in Preadolescent Students: A Cross Sectional Study
Source: Front Psychol. 2019 Jun 12;10:1287. doi: 10.3389/fpsyg.2019.01287 (PMC6598732; doi:10.3389/fpsyg.2019.01287)
Supplement: Supplementary file 1 [file Data_Sheet_1.PDF]

Table 1

Individual characteristics and risk behaviors: gamblers versus nongamblers

	Gamblers n = 379	Nongamblers n = 2096	Test value	p-value
Gender, male	288 (76)[a]	1024 (48.9)	$\chi^2 = 94.87$	< .001
Inappropriate school behavior	146 (38.5)	367 (17.5)	$\chi^2 = 86.25$	< .001
Parents with gambling behavior	183 (57.5)	135 (42.5)	$\chi^2 = 179.2$	< .001
Troubles with parents - gambling related	16 (4.2)	19 (0.9)	$\chi^2 = 25.3$	< .001
Troubles with parents – videogames related	165 (43.7)	471 (22.6)	$\chi^2 = 74.1$	< .001
Online gambling without money	136 (36.1)	205 (9.9)	$\chi^2 = 182.4$	< .001
Age	12.69 ± 0.95[b]	12.29 ± 0.89	$t = 7.99$	< .001

Test value: $\chi^2 = \text{Pearson chi} - \text{square}$; $t = t - \text{test}$;

[a] n (%).

[b] Mean $\pm$ SD.

[c] Equal variances not assumed.
