# Supplementary material for: Gambling Behavior and Risk Factors in Preadolescent Students: A Cross Sectional Study
Source: Front Psychol. 2019 Jun 12;10:1287. doi: 10.3389/fpsyg.2019.01287 (PMC6598732; doi:10.3389/fpsyg.2019.01287)
Supplement: Supplementary file 2 [file Data_Sheet_2.PDF]

Table 2

Logistic regression predicting likelihood of gambling behavior in preadolescents

	B	SE	Wald	*df*	*p*	Odds Ratio	95% C.I. for Odds Ratio	
							Lower	Upper
Gender (Male)	-1.09	.16	48.08	1	.000	.34	.25	.46
Inappropriate school behavior (No)	.62	.15	17.93	1	.000	1.86	1.39	2.48
Parents with gambling behavior (No)	1.34	.15	82.68	1	.000	3.84	2.87	5.13
Troubles with parents - gambling related (No)	.25	.47	.28	1	.595	1.28	.51	3.24
Troubles with parents – videogames related (No)	.29	.15	3.85	1	.050	1.34	1.00	1.78
Online gambling without money (No)	.83	.16	27.27	1	.000	2.30	1.68	3.14
Age	.34	.07	22.68	1	.000	1.40	1.22	1.61

Note: In parentheses, the reference category.
