# Supplementary material for: Gambling Behavior and Risk Factors in Preadolescent Students: A Cross Sectional Study
Source: Front Psychol. 2019 Jun 12;10:1287. doi: 10.3389/fpsyg.2019.01287 (PMC6598732; doi:10.3389/fpsyg.2019.01287)
Supplement: Supplementary file 3 [file Data_Sheet_3.PDF]

Table 3

Friedman test - Pairwise comparisons of sample average ranks

	Test Statistic	SE	Std. Test Statistic	Sig.	Adj. Sig.
Videopoker, Slot Machines, Video Slots – Lotto, lottery, SuperJackpot	.048	.116	.417	.676	1
Videopoker, Slot Machines, Video Slots – Scratch card	.840	.116	7.246	.000	.000
Videopoker, Slot Machines, Video Slots – Sport bets	.892	.116	7.698	.000	.000
Videopoker, Slot Machines, Video Slots – Daily fantasy sports	.941	.116	8.115	.000	.000
Lotto, lottery, SuperJackpot – Scratch card	.792	.116	6.829	.000	.000
Lotto, lottery, SuperJackpot – Sport bets	- .844	.116	- 7.281	.000	.000
Lotto, lottery, SuperJackpot – Daily fantasy sports	- .892	.116	- 7.698	.000	.000
Scratch card – Sport bets	- .052	.116	- .452	.651	1
Scratch card – Daily fantasy sports	- .101	.116	- .870	.385	1
Sport bets – Scratch card	.048	.116	.417	.676	1
