# Supplementary material for: Gambling Behavior and Risk Factors in Preadolescent Students: A Cross Sectional Study
Source: Front Psychol. 2019 Jun 12;10:1287. doi: 10.3389/fpsyg.2019.01287 (PMC6598732; doi:10.3389/fpsyg.2019.01287)
Supplement: Supplementary file 4 [file Data_Sheet_4.PDF]

Gambling Behavior and risk factors in pre adolescent students:

a cross sectional study

Nicoletta Vegni[1*], Francesco Maria Melchiori[1], Claudia Prestano[1], Caterina D'Ardia[1], Massimo Canu[1], Giulia Piergiovanni[1], and Gloria Di Filippo[1]

QUESTIONNAIRE EXCERPT

2. Gender
❍ F ❍ M

3. Age

6. Have you ever had problems with school behaviour in the last twelve months (notes, reports, suspensions)?
❍ yes ❍ No

The following questions concern Gambling

Gambling consists of betting goods, such as money, on the outcome of a future event (e.g. the outcome of a football match).
The most popular games and bets are: Scratch cards, Lotto, lottery, SuperJackpot, Videopoker, Bingo, card games, Sport bets, Fantasy football, Slot Machine, games and bets on the Internet.
With some games on Internet you can also play without money (e.g. Slot Machine or Video Slot).

7. In the last 12 months, you've been gambling and betting money?
❍ yes ❍ No

8. In the last 12 months, how often have you played the following games?

	Never	Less than once a month	Monthly	Several times a week
Scratch card	❍	❍	❍	❍
Lotto, lottery, SuperJackpot	❍	❍	❍	❍
Daily fantasy sports	❍	❍	❍	❍
Sport bets	❍	❍	❍	❍
Videopoker, Slot Machine, Video Slot	❍	❍	❍	❍

9. Do your parents play or bet money?

❍ yes ❍ No ❍ I don't know

10. What do you think about gambling?

	Completely agree	Agree	Neither agree or disagree	Disagree	Completely disagree
It is funny	☐	☐	☐	☐	☐
It is risky	☐	☐	☐	☐	☐
It is exciting	☐	☐	☐	☐	☐
It can become a habit	☐	☐	☐	☐	☐
You lose money	☐	☐	☐	☐	☐
You become rich	☐	☐	☐	☐	☐

13. Have you ever gambled on the Internet with money (using computers, smartphones or tablets)?

❍ yes ❍ No

15. In the last 12 months, have you ever had problems with your parents because of Internet gambling?

❍ yes ❍ No

18. In the last 12 months, has playing video games caused you to have family discussions?

❍ Yes ❍ No
